# Consensus Recommendations for Prevention of Nutritional Rickets: Food Fortification and Micronutrient Supplements for Global Health

**DOI:** 10.3934/publichealth.2016.1.40

**Published:** 2016-01-29

**Authors:** Wolfgang Högler, Magda Aguiar, Mairead Kiely, Theodore Tulchinsky

**Affiliations:** 1 Department of Endocrinology & Diabetes, Birmingham Children's Hospital; and Institute of Metabolism and Systems Research, University of Birmingham, Birmingham, United Kingdom; 2 Department of Health Economics, Institute of Applied Health Research, University of Birmingham, United Kingdom; 3 Vitamin D Research Group, School of Food and Nutritional Sciences, University College Cork, Ireland; 4 Professor Emeritus: Braun School of Public Health and Community Medicine, Hebrew University-Hadassah, 91220 Jerusalem, Israel. Head of School of Health Professions, Ashkelon College, Ashkelon, Israel

**Keywords:** Food fortification, micronutrient deficiencies (MNDs), vitamin D, rickets, folic acid, iron, iodine, vitamin B complex, prevention

*Malnutrition*, in its many forms, is a problem of staggering size - large enough to threaten the world's sustainable development ambitions [1]. Tackling malnutrition is critical for social and economic development, and the Global Nutrition Report reported encouraging progress in several countries [2]. However, slow or no progress has been made on addressing the persistent problem of micronutrient deficiencies (MNDs), with limited data for coverage of micronutrient-specific interventions, including folic acid supplementation or fortification, universal salt iodization, supplementation of calcium, vitamin A, multiple micronutrients and zinc, as well as zinc treatment for diarrhea [3]–[5].

MND's are underlying factors in the pathogenesis of acute and chronic disease, including developmental disorders and non-communicable chronic diseases, and hinder achievement of international health goals for maternal and child health but also for education and other defined targets [2]. The World Health Organization (WHO) estimates that around 2 billion people suffer from MNDs across a wide spectrum of societies in all countries [2]–[5]. Some essential micronutrients have been addressed in the global health community but others, such as vitamin D, have been neglected [6].

As representatives of a Global Consensus Group, we advocate for the eradication of nutritional rickets and osteomalacia caused by Vitamin D deficiency and/or dietary calcium deficiency. These deficiencies represent a global health pandemic [6],[7], a problem of the first magnitude for global health. We recognize that MNDs do not necessarily occur in isolation, so we consider Vitamin D deficiency in a wider context of other important deficiencies including iron, iodine and vitamin B complex which are prioritized by the WHO.

The *recommendations of the global consensus for the prevention and management of nutritional rickets* have recently been co-published in the Journal of Clinical Endocrinology and Metabolism [8] and Hormone Research in Paediatrics [9]. The consensus group consisted of international experts in paediatric endocrinology and bone, paediatrics, nutrition, epidemiology, health economics and public health. Each participant performed a systematic literature review on a particular consensus question and graded the quality of the evidence according to the GRADE method [10]. This process was followed by a 3-day consultation meeting in Birmingham, in the spring of 2014 where five working groups, divided across the topics of diagnosis and treatment of nutritional rickets, risk characterization (exposure), pregnancy and lactation and public health recommendations, summarized the main outcomes of each of the 33 consensus questions.

After presenting and debating the evidence and allowing freedom of expression of differing views, we reached the unanimous conclusion that this topic requires urgent reassessment by the major national and international health stakeholders. We concluded that nutritional rickets and osteomalacia, secondary to calcium and/or vitamin D deficiency, are a major preventable global health problem [4]–[7], not confined to high latitude, social class or age groups. Rickets and osteomalacia are just as apparent in Africa, Asia and the Middle East, as in risk groups living in Europe, Australasia and the Americas [11]–[19]. In the case of vitamin D, risk groups constitute mainly individuals with dark skin or those wearing traditional full-body covers [6]–[9],[11]–[19].

*Morbidity and mortality from prolonged and severe vitamin D deficiency* should not be underestimated. The burden of disease includes hypocalcaemic seizures, hypocalcaemic dilated cardiomyopathy with heart failure, muscle weakness, growth failure, rickets and osteomalacia. Complications include pain, fractures, bone deformity and long-term disability, obstructed labor, increased risk of falls and death [8],[9],[20]. Chronic disease groups, especially the elderly, are also affected from under-recognized muscle weakness, osteomalacia and associated falls and fractures.

Since rickets and osteomalacia are fully preventable, we advocate for their eradication through implementation of international supplementation and food fortification programs containing vitamin D and/or calcium. Based on high quality evidence, our consensus group issued strong recommendation to provide vitamin D supplementation to: 1) all infants from birth to at least 12 months of age; 2) all pregnant women; and 3) all individuals from high risk groups. Supplementation works best when integrated into public health programs alongside immunization and antenatal care programs [8],[9].

We acknowledge that, according to the setting and the targeted populations, *supplementation may face significant challenges*, namely low uptake rates, difficulties in reaching remote populations, inappropriate legislation and infrastructures and/or insufficiently skilled professionals to provide an effective distribution chain. For example, substantial differences exist in uptake of infant vitamin D supplementation in Europe [20]. Food fortification is a viable alternative supportive program to reach the whole population [11]–[21]. Fortification is generally easy to implement and cost-effective as the distribution of the micronutrient relies on a distribution chain already established by the food industry. In addition, no additional burden is posed on the health system as fortification is not dependent on uptake rates and, if the vehicle selected is adequate, provides wide coverage.

The most established instrument for national authorities to implement preventative recommendations is *fortification of commodity or habitually consumed foods* such as milk, cooking oils or other vehicles as appropriate in each country [22]–[26]. The public health decision of fortification should not be left to the manufacturers but be policy-driven and monitored by public authorities [27],[28]. Food fortification has been basic public health practice in Canada and the United States for decades, as well as many other countries across the world. Evidence confirms that this practice is safe, cost-effective and acceptable to manufacturers as well as the public generally. Such fortification has successfully prevented specific diseases, including rickets and birth defects such as neural tube defects [22],[28],[29]. However, such fortification strategies require governmental leadership with supportive legislation, monitoring and, most important, adequate selection of the vehicle, ideally a staple food, consumed regularly by risk groups in predictable amounts [21],[22],[28].

**Figure 1.**
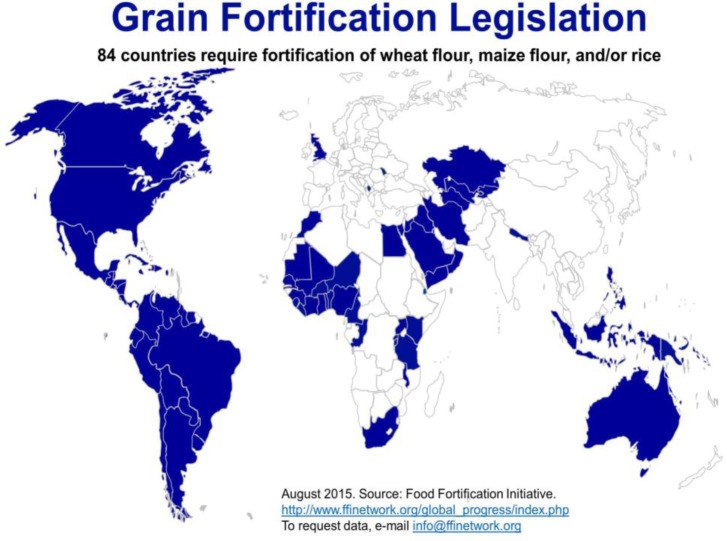
Countries Which Adopted Mandatory Fortification of Wheat and Maize Flour and/or Rice. Source: Food Fortification Initiative. Food control and monitoring. Website: Available at: http://www.ffinetwork.org/global_progress/LegislationAugust2015.jpg (Accessed 27 October 2015).

Similar to nutritional rickets, *folic acid deficiency* has been somewhat neglected as a preventable MND, despite great success in food fortification programs. Folic acid supplements were introduced for women of childbearing age in the 1990s to prevent neural tube defects (NTDs). But supplementation was found to cover only one third of the target population. As a result, folic acid was added to flour in Canada, the United States and Chile in 1998 and subsequently in all countries in Central/South America, Australia, sub-Saharan Africa and many others [26–28;30–32]. ***Figure 1*** shows which countries (in blue) have adopted mandatory fortification of flour or rice with folic acid, mostly along with iron and vitamin B complex [25]. Addressing multiple deficiencies with food fortification is a safe methodology, relatively easy to implement, regulate and monitor as a cost-effective and sustainable strategy.

The Food and Agriculture Organization states: “Food fortification is an essential element in nutrition strategies to alleviate micronutrient deficiencies. It is a dynamic area developing in response to the needs of population groups and industry. Efforts should continue to develop improved and new systems of delivering micronutrients to target populations through appropriate fortification procedures.” [26].

The effectiveness of folic acid fortification of flour in reducing incidence of NTDs has been well demonstrated [33]–[35]. For example, since mandatory flour fortification with folic acid was introduced in Canada in 1998, the overall rate of NTDs has decreased by 46%, including live births, stillbirths and cases detected prenatally in pregnancies that were subsequently terminated” [35]. In contrast, there has been no decline in NTDs in 19 European countries from 1991 to 2011, since no mandatory food fortification is in place [36]. This global inequality persists despite longstanding European recommendations aimed at promoting peri-conceptional folic acid supplementation and the existence of voluntary folic acid fortification policies. The European nations will have to consider putting legislation in place to make fortification of staple foods with folic acid (and other micronutrients) mandatory.

*Iron deficiency (ID)* is the most common MND worldwide, particularly in low-income countries and high-risk populations [37]. Iron deficiency anemia (IDA) is responsible for 20 percent of all maternal mortality and contributes to poor physical and mental development in children. Risk groups for IDA are pregnant women and young children, specifically those with low birth weight, high cow's-milk intake, low intake of iron-rich complementary foods, low socioeconomic status, immigrant status and the chronically ill [38]. This risk profile for IDA therefore has lots of similarities with that for rickets. Iron fortification of formula milk and wheat flour is practiced efficiently worldwide with millions of person years of exposure [39]. In Europe, ESPGHAN found insufficient evidence to support general iron supplementation of healthy European infants of normal birth weight [38], whilst highlighting high-risk groups for supplementation. A potential risk of worsening undiagnosed congenital haemochromatosis has not been substantiated to date by evidence, and flour fortification with iron in developing countries is generally considered safe [40]–[44].

Rickets Prevention by Supplementation, Fortification or Both?

Since supplementation targets individuals only, requires compliance and is more costly, we consider food fortification programs (vitamin D and/or calcium) to be most feasible, efficient and cheaper for eradication of rickets since it raises vitamin D and calcium status of the whole population [22];[30]–[32], along with other essential micronutrients. Food fortification and supplementation programs should be seen as synergetic interventions, i.e. the optimal nutrition status of the population is more likely to be reached when food fortification is complemented with supplementation of risk groups such as infants and pregnant women [45].

*Global health initiatives* have helped to advance health in low-income countries most dramatically through immunization programs for eradication of smallpox, polio, measles, rubella and many other vaccine-preventable diseases. These activities have contributed enormously to reducing child mortality. Success is also being achieved in public health control minimizing tropical diseases such as leprosy and onchocerciasis [2]. National governments, international donors and stakeholders such as the WHO, the World Bank, UNDP, UNICEF, the Bill and Melinda Gates Foundation, GAVI, the Rotary Club and others contributed greatly to achievements in the Millennium Development Goals. Although our Consensus Group focused on its mandate of prevention of rickets it is important to stress that MNDs often occur as combined deficiencies and are responsible for multiple assaults on the physiology and wellbeing of many groups in any population.

MNDs are particularly serious in young children and the elderly as they increase mortality from relatively minor conditions, such as diarrheal or respiratory infections, which would not negatively impact a well nourished person, as demonstrated by the association of vitamin A deficiency and high case fatality rates in children from measles. We recommend that prevention of MNDs, including vitamin D deficiency, be added to the list of successful and promoted activities. This is in line with conclusions of the Copenhagen Consensus Centre 2008, formed by a group of leading economists that ranked *micronutrient supplementation and fortification among the top three international development priorities*. The top ranking is due to their highly favorable cost-benefit ratios compared to other intervention such as expanding immunization (ranked 4^th^), malaria prevention and treatment (ranked 12^th^) and HIV combination prevention (ranked 19^th^) [46].

*In conclusion*, we call on the international agencies for health and social development to coordinate efforts and work with national states and regional bodies such as the European Union and political networks of African countries to make the eradication of micronutrient malnutrition a high priority for the coming decades. The United Nations Sustainable Development Goals for the year 2030 includes reducing hunger, overt and covert malnutrition, neonatal mortality among other goals for food security and modern agriculture [47].

In the context of wider public health policies placing MNDs as a high priority public health challenge, we specifically call on national and international health agencies to place high priority on elimination of the global pandemic of rickets and osteomalacia by implementing the following measures [8],[9]:

1. Establish provision of daily vitamin D supplements to all infants from birth to at least 12 months of age (400 International units (IU)/day), regardless of mode of feeding as part of routine child care;
2. Establish provision of daily vitamin D supplements to all pregnant women (600 IU/day) ideally in preparations combined with folic acid and iron.
3. Ensure that all women of reproductive age meet their nutritional requirements of vitamin D (600 IU/day), either through diet or supplements.
4. Establish provision of daily vitamin D supplements (600 IU/day) to all risk groups.
5. Implement fortification of staple foods such as milk, cooking oils or flour with vitamin D in order to raise the levels of vitamin D at population levels.

Further, we call on national and international agencies to expedite implementation of mandatory fortification of salt with iodine, and flour (wheat, maize and corn) with folic acid, vitamin B complex and, specific to developing countries, also iron.
